# Ionizing Radiation and Complex DNA Damage: Quantifying the Radiobiological Damage Using Monte Carlo Simulations

**DOI:** 10.3390/cancers12040799

**Published:** 2020-03-26

**Authors:** Konstantinos P. Chatzipapas, Panagiotis Papadimitroulas, Dimitris Emfietzoglou, Spyridon A. Kalospyros, Megumi Hada, Alexandros G. Georgakilas, George C. Kagadis

**Affiliations:** 1 3dmi Research Group, Department of Medical Physics, School of Medicine, University of Patras, 26504 Rion, Greece; kwnchatz@upatras.gr; 2 Bioemission Technology Solutions (BIOEMTECH), 11472 Athens, Greece; panpap@bioemtech.com; 3 Department of Medical Physics, School of Medicine, University of Ioannina, 45500 Ioannina, Greece; demfietz@gmail.com; 4 DNA Damage Laboratory, Department of Physics, School of Applied Mathematical and Physical Sciences, National Technical University of Athens (NTUA), 15780 Athens, Greece; spyrcals@gmail.com (S.A.K.); alexg@mail.ntua.gr (A.G.G.); 5 Radiation Institute for Science & Engineering, Prairie View A&M University, Prairie View, TX 77446, USA; mehada@pvamu.edu

**Keywords:** radiobiology, nanoscale simulations, Monte Carlo method, ionizing radiation, complex DNA damage, biological response

## Abstract

Ionizing radiation is a common tool in medical procedures. Monte Carlo (MC) techniques are widely used when dosimetry is the matter of investigation. The scientific community has invested, over the last 20 years, a lot of effort into improving the knowledge of radiation biology. The present article aims to summarize the understanding of the field of DNA damage response (DDR) to ionizing radiation by providing an overview on MC simulation studies that try to explain several aspects of radiation biology. The need for accurate techniques for the quantification of DNA damage is crucial, as it becomes a clinical need to evaluate the outcome of various applications including both low- and high-energy radiation medical procedures. Understanding DNA repair processes would improve radiation therapy procedures. Monte Carlo simulations are a promising tool in radiobiology studies, as there are clear prospects for more advanced tools that could be used in multidisciplinary studies, in the fields of physics, medicine, biology and chemistry. Still, lot of effort is needed to evolve MC simulation tools and apply them in multiscale studies starting from small DNA segments and reaching a population of cells.

## 1. Introduction

Ionizing radiation (IR) is used in several medical procedures either for therapeutic and/or for imaging/diagnostic procedures. When used for therapeutic purposes, e.g., to effectively treat cancer, the main goal is to successfully irradiate malignant cells while ensuring healthy tissues absorb the lowest possible dose. Following this technique, malignant cells are killed while healthy cells remain healthy, avoiding possible genetic aberrations or cell death [1,2,3,4]. To achieve this objective, it is essential to accurately predict the consequences following IR interaction with biological matter. Accidental, natural, and occupational exposure to IR must not been overlooked because of their possible great biological impact. 

The scientific community has invested a lot of effort in understanding the response mechanisms initiated and generally involved in IR-induced DNA damage. Particularly, the exact radiochemical mechanisms that produce single strand breaks (SSBs), double strand breaks (DSBs), and clustered/complex DNA damage (CDD) which can consist of multiple DSBs and/or closely spaced (within 10–20 base pairs) non-DSB lesions, such as oxidized bases and abasic sites [5,6], as well as the DNA damage response (DDR) and repair pathways activated through the whole procedure have been investigated. The great variation of pathways that actively participate in ‘response-to-radiation’ prompts extensive investigation. It has to be mentioned that the aforementioned processes could lead to oncogenic transformations, but the achievement of wider knowledge will guide the scientific community to the biological optimization of radiation therapy for cancer treatment [7,8,9,10,11]. 

The present review article aims to improve the understanding of the field of IR-DDR in general by providing an overview of Monte Carlo simulation studies that try to explain several aspects of cancer treatment using IR. Some mathematical studies on the investigation of DDR have also been included.

**Figure 1:**
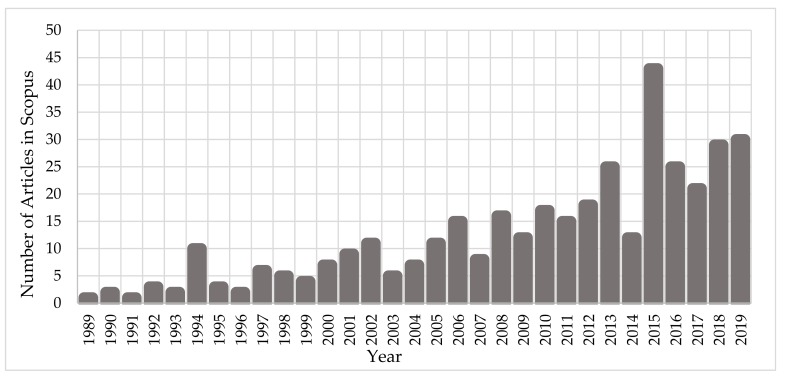
Number of published articles, over the last 30 years, on “Monte Carlo simulations” for the investigation of “DNA damage”.

Since the early ‘90s, the scientific community has been investigating the interaction mechanisms of IR on cells and DNA. Figure 1 depicts the growth of the number of published scientific studies in the field of radiobiology using Monte Carlo techniques. A large increase in the number of studies is observed from 2010, coinciding with the great evolution in computer science and advanced computational tools for Monte Carlo simulations on the molecular and cellular level. According to Scopus (www.scopus.com), the relevant documents in “DNA damage” and “Monte Carlo simulations” total almost 380 over the last 30 years (Figure 1), showing the emerging field of computational radiobiology. This review aims to summarize the studies that were performed on the DNA modelling for Monte Carlo simulations, on the DDR, and on the DNA repair mechanisms. 

## 2. An Overview of the Methods Used in Nanoscale Simulations

Monte Carlo techniques take advantage of random number generators and computers to simulate complex systems that are not easy to approach in an analytical way. Monte Carlo methods originate from innovative work by Ulam, von Neumann, Metropolis and Fermi in the 1930s and 1940s [12,13], and are described nicely in Andrieu et al. [14]. At later stages, MC techniques were used by Eckhardt [15] and since then they have been applied in many disciplines. They are used in board games, weather forecasting, electrical and telecommunication engineering, quantum physics, and computational biology [16]. In medical physics, they are used to simulate a range of medical applications that exploit IR, for imaging purposes or for dosimetry applications on the macro- and nano-scale [17]. 

We should mention the fact that to be able to have absolutely comparable information on the output of each MC code, data shall be given on alternative approaches, definitely including particle types and energies for which the code is applicable, underlying cross sections, assumptions on radiochemistry, whether indirect damage is included at all, and how DNA and chromatin structures are modelled. At present, information provided on distinct codes is of a very heterogeneous nature. For instance, simulations on the effects of gold nanoparticles are mentioned with Geant4-DNA only, but these have been performed with other codes as well, cf. the recent review by Li et al Phys Med 2020 [18].

Pioneers to MC simulations of DNA energy deposition and strand breaks were Paretzke H.G., Goodhead D.T., Nikjoo H. and Tomita H. In 1989, Nikjoo et al. [19] published a study (based on [20]) where they used virtual cylindrical geometrical models to imitate biological models of DNA segments, nucleosomes, and chromatin fibers. To determine the energy deposition in a target volume by ultra-soft X-rays they used the already published, in 1988, mathematical model for track structure analysis by Goodhead et al. [21]. Tomita et al. [22] went a step further and used monoenergetic electrons and new DNA structure models (one turn of double-strand DNA, nucleosome, solenoid) to study physical and chemical stages of the radiation interaction. They studied both direct and indirect effects of radiation on the DNA.

### 2.1. Particle Track Structure Codes

Monte Carlo track structure (MCTS) codes are widely used for simulating the transportation of ionizing particles in biological matter at small scales (nm-μm). Thus, they offer a valuable theoretical tool for mechanistic radiation effect studies, especially in quantifying DNA damage under different radiation qualities, which still presents a major challenge in radiobiology research [23]. This is not possible with most general-purpose MC radiation transport codes such as MCNP [24], EGS [25], FLUKA [26], PENELOPE [27], and PHITS [28], which are widely used for dosimetry at the tissue and organ level (~mm-cm scale). The limitation of these codes stems from the use of so-called condensed-history physics models [29]. The essence of these models is the grouping of a large number of interactions along artificial steps, which limits their resolution to about 10–100 μm (at best), and prohibits their application at low energies (below about 1–10 keV) [30]. 

**Figure 2:**
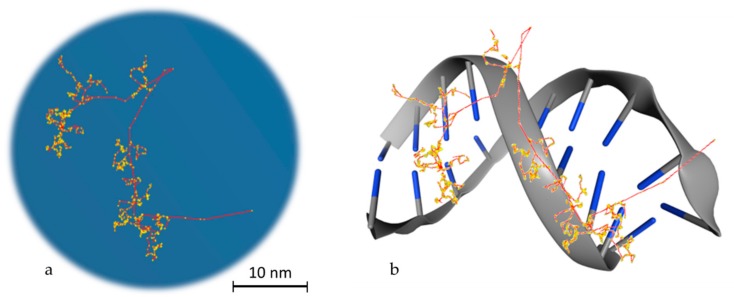
Particle transport example. (**a**) Track structure of an electron (10 KeV) in water as simulated in Geant4-DNA. The red line, which starts from the bottom right edge, is the route followed by the primary electron, while in yellow the interactions with the water medium are presented. The red branches that are separated from the main route represent secondary electrons. (**b**) Schematic representation of the way that energy is deposited on DNA molecules. This is a stylish representation, which has the aim of helping the reader understand the way that the superposition of energy deposition in water is transformed to single or double strand breaks. The blue edges imitate DNA bases.

To reach a spatial resolution at the DNA level, it is necessary to adopt a more detailed description of particle transport, as shown in Figure 2. This is achieved by simulating all elastic and inelastic collisions one-by-one, until all particles slow down to thermalization energies or, in practice, the ionizing threshold of the medium (which is in around 10 eV for tissue-like materials). The aim is to generate a three-dimensional map of the radiation track based on the spatial distribution of energy-transfer points in the medium [31]. This functionality has provided a unique theoretical tool for investigating differences between sparse (or low linear energy transfer (LET)) and dense (or high-LET) ionization radiations at the nanometer level, which is not possible by other means [32]. MCTS codes have been the workhorse of theoretical microdosimetry, enabling systematic calculations of lineal energy spectra (the stochastic analog of LET), proximity functions, ionization cluster distributions, etc., which are used for explaining and predicting the quality factor or the relative biological effectiveness of different ionizing radiations [33,34,35,36,37,38,39]. In addition, quantitative estimates of the early “direct” DNA damage can be obtained by combining the spatial distribution of energy-transfer points (above a certain threshold) with the geometric structure of DNA [40,41,42,43,44,45]. The main drawback of MCTS codes is that they are computer intensive and require much more detailed physics models than currently available ones [46]. In particular, track-structure models for low-energy transport must strongly rely on theory since experimental data are scarce, and they are highly material-specific which limits their application range [47]. Thus, MCTS simulations are usually application-specific and are developed for a single media, most commonly water. 

MCTS codes for water have been used for over 40 years in radiation biophysics, and more than a dozen such codes have been reported in the literature [23], with the most widely used being NOREC [48], PARTRAC [20], KURBUC [49], and RITRACKS [50]. Although most of the early MCTS codes were limited to electrons, nowadays most MCTS codes can also simulate more heavily charged particles like protons, alphas, and some ions. NOREC (NIST-modified Oak Ridge Electron Code) is an upgraded version of the OREC code developed in the ‘70s at the Oak Ridge National Laboratory (ORNL) by Ritchie and co-workers [51]. OREC was the first MCTS code to implement a realistic physics model of the liquid-phase of water based on an empirical dielectric response function deduced from optical measurements at ORNL [52]. This major achievement allowed the calculation of ionization and excitation cross sections for condensed-phase water from first principles. OREC was also one of the first MCTS codes to extend the simulations to the chemical stage with a detailed radiolysis model that was extensively benchmarked against experimental data [53]. In the early 2000s, following an upgrade of the elastic scattering model based on NIST’s ELAST database, OREC was renamed to NOREC [48]. NOREC can simulate full slowing-down electron tracks over the energy range from 7.4 eV to 1 MeV. PARTRAC (PARticle TRACks) is an evolution of the MOCA (MOnte-CArlo) code developed in the 1970s by Paretzke and co-workers at the National Research Center for Environment and Health (GSF) of Germany. MOCA was one of the first MCTS codes for electron transport in biological matter represented by unit-density gaseous water [20]. PARTRAC has evolved to a comprehensive MCTS code that extends to the chemical and biological stage, including multi-scale models for the structure of the whole genomic DNA with atomic resolution, which are supplemented with DNA damage and repair models [54]. It has also adopted more realistic physics models for the biological medium based on the empirical dielectric response function of liquid water developed by Dingfelder and co-workers [55]. PARTRAC can transport electrons (10 eV–10 MeV), protons (1 keV–1 GeV), alpha-particles (1 keV–1 GeV), and light ions (from <1 MeV/u up to 1 GeV/u) [56], and it can also take into account water radiolysis, DNA repair and chromosomal aberrations [57,58,59]. KURBUC (Kyushu University and Radiation Biophysics Unit Code) was initially developed at the Medical Research Council (MRC) of UK in the early 1990s by Nikjoo and co-workers using mostly empirical and parameterized physics models for electron transport in unit-density gaseous water mediums in the energy range from 10 eV to 10 MeV [60]. It was later extended to protons (1 keV–1 MeV), alpha particles (1 keV/u–2 MeV/u), neutrons (thermal to 100 MeV), and carbon ions (1 keV/u–10 MeV/u). In its latest version, KURBUCliq [49], a more realistic physics model for biological matter, has been implemented using the dielectric response function developed by Emfietzoglou, Cucinotta, and Nikjoo (known as the ECN model) [61] which is based on the latest experimental data for liquid water. KURBUCliq can also perform simulations of the chemical and biological stage including sophisticated mathematical models of DNA damage and repair pathways along with atomistic DNA structure models [62,63,64]. RITRACKS (Relativistic Ion TRACKS) is a NASA-funded MCTS code that extends the transport of ionizing particles in unit-density gaseous water mediums up to the very highest energies (100 MeV for electrons and 10 GeV/u for ions), which are of interest to space radiation protection. Molecular cross sections specific to the DNA bases (in the gas phase) are also available towards a more realistic approximation of direct ionizations in DNA. A distinct feature is the implementation of time-dependent track effects in order to simulate dose-rate effects. It also includes modeling of water radiolysis and simple DNA structures for radiation damage studies [65]. Recently, the RITRACKS code has been used and the results advocate for the hypothesis that not only a single particle may induce many non-DSB clustered damages, but also that there may be a large number of them in a chromatin fiber [66]. The TRAX code performs simulations for track-structure applications in various media [67]. MC4 performs simulations down to very low energies, including models for gaseous and liquid water, and is known for its upgraded models for the liquid phase [68,69]. PHITS has been developed to simulate the track structure of electrons in liquid water in a wide incident energy spectrum from 1 meV to 1 MeV [70].

The aforementioned MCTS codes are not publicly available. Therefore, in recent years, there have been efforts to implement track-structure models into some general-purpose MC codes to enable simulations at both the macro and microscopic scale. The most notable examples are the Monte Carlo N-Particle version 6 (MCNP6) [71], PENELOPE (modification to PENELOPE/penEasy, resulting in the LionTrack code) [72,73,74], and the Geant4-DNA package of Geant4 [75,76,77]. Apart from MCNP6, which uses a simple interpolation of high-energy atomic models down to the eV energy range, thus neglecting molecular aggregation and condensed-phase effects, the Geant4-DNA and modified PENELOPE/penEasy extensions are based on elaborate physics models specifically developed for liquid water. The code PENELOPE is known for its electron models and extends accurately to low energies, having applications to micro- and nano-dosimetry [78,79], however, the low-energy modification of the PENELOPE code is not publicly distributed. Therefore, since 2007, Geant4 (release 9.1) is the only open access general-purpose radiation transport MC code offering, through its Geant4-DNA extension, track-structure functionalities in liquid water down to the eV energy range for radiobiological applications at the cellular, sub-cellular, and DNA level [75,76]. Due to the prominent role of low-energy electrons (called “track-ends”) in radiation-induced DNA damage, a distinct feature of Geant4-DNA is the availability of different physics models for the interaction of low-energy electrons with liquid water [39,80,81,82]. These models, which adopt either the dielectric response function approach or other hybrid approaches, undergo continuous development and refinement. Recently, track-structure models for gold nanoparticles have also been developed and will be incorporated in the upcoming Geant4-DNA releases [83,84,85]. It also includes modelling of the chemical stage [86] and offers various representations of DNA target structures to be used in DNA damage studies [87,88,89]. Geant4-DNA can transport electrons (7.4 eV–1 MeV), protons (100 eV–100 MeV), alpha particles (1 keV–400 MeV), and ions (0.5 MeV/u–10^6^ MeV/u). It is noteworthy that the TOPAS-nBio software [90], which extends the TOPAS MC code [91] to the (sub) cellular and DNA scale for radiobiological studies [92], is based on the Geant4-DNA package.

The aforementioned MCTS codes have great potential to expand the knowledge of mechanisms that are involved in the response of biological matter to IR, but they also have limitations. The limitations vary from the lack of experimental information on the processes involved, which are also under research, up to the broad uncertainty of used parameters or the large fluctuations of parameters that are exploited to describe cell sensitivity. Another obstacle is that not all MCTS codes are benchmarked, especially with respect to nanodosimetric calculations. MCTS codes are very sensitive to the physics cross sections that are used for particle transportation as they are the core of the computation. These cross sections vary depending on how they are produced (analytically, extrapolation, or experimentally) and therefore they introduce a wide uncertainty to the simulation of track structures. This undoubtedly influences the results of MCTS simulations, e.g., the number of interactions within a certain volume, which is very critical when it comes to nanodosimetry (somewhat less for microdosimety). Thus, there is an urgent need for benchmarking MCTS codes with some efforts being done recently [93]. The computational time of the simulation for a population of cells is also a big concern. MCTS codes are also not capable enough to reliably simulate the very low energy secondary electrons below about 10 eV, which are considered as potential lethal sources for the cell since they can induce harmful clustered lesions in the DNA [94,95].

### 2.2. Monte Carlo Techniques for Radiobiological Modelling

Currently, there are two methods used to estimate DNA damage. The first method is to estimate potential DSBs by superimposing DNA geometry to the radiation track structure [42,44,96,97,98]. The other method is to use clustering algorithms based on probabilistic models to estimate DSB yield [99,100,101]. The first method is more direct way to estimate DNA damage accurately, but it is also a time-consuming procedure. The second method can also calculate with reasonable accuracy the DNA DSB, and it reduces the computational time. Monte Carlo Damage Simulation (MCDS) [102,103,104] and DBSCAN [105] are two platforms that can be used for such a purpose. A study has already been done to compare DBSCAN with Geant4-DNA simulation results by Lampe et al. [106,107].

The RADAMOL [108,109] toolbox includes the simulation of chemical and physico-chemical stages produced when ionizing particles interact with water. To simulate scavengeable and unscavengeable DNA damage, it also includes RADACK and DIRADACK. PARTRAC [55] is focused on radiation biology and can simulate both physics and chemistry interactions produced by the irradiation of biological materials. PARTRAC also includes mathematical models of biology to study the procedure of recombination of DNA segments after irradiation [110] and has integrated information on DNA and chromatin structure over multiple levels and the susceptibility of DNA damage, as well as enzymatic reactions that aim to restore the integrity of the cellular DNA. PARTRAC, in its follow-up chemistry component, can account for inter-track effects [111], a feature that Geant4-DNA also implanted in its code. Geant4-DNA has been often compared with PARTRAC, for validation purposes [112,113], while PARTRAC publications influenced the design of Geant4-DNA code.

A recent addition on simulating radiation chemistry and track structures at the subcellular scale is the MPEXS-DNA, GPU-based Monte Carlo simulator [114]. MPEXS-DNA uses the speed of GPU processing for the physical, physicochemical, chemical and biological stages of the simulation process of IR interaction with biological matter. It is based on the Geant4-DNA package and can also simulate chemical reactions and the diffusion process for molecular species, and the distribution of molecular species can also be determined. In this study [114], the authors confirmed that the simulation results, which were obtained using MPEXS-DNA, were consistent with existing experimental and simulation data. Compared with simulations that were performed in Geant4-DNA with a single CPU core, MPEXSDNA performed the same simulations thousands times faster, keeping the accuracy at the same level.

The Monte Carlo Damage Simulation (MCDS) [102,103] algorithm, as a cell-level model, is a quasi-phenomenological concept used to simulate DNA damage yields (SSBs, DSBs and sites of multiple base damage, average number of lesions per DNA damage cluster, as well as cluster length in base pairs). It is a fast algorithm compared to common track-structure simulations (execution time ranges from seconds to some minutes on a typical PC). It utilizes monoenergetic electrons, protons, α-particles and other charged particles with atomic numbers up to Z = 26 (i.e., ^56^Fe), with kinetic energies up to a few GeV [102,103,104]. It also has the ability to simulate damage induction for random mixtures of charged particles. For photons and neutrons, MCDS can provide the distribution of secondary charged particles produced via the interaction of neutral particles with a target region of interest [115]. Considering the spectrum of energy used for the particles involved in MCDS, the minimum allowed kinetic energy limit depends on the particle type: e.g., for electrons, damage induction can be simulated from some tens of eV, while for bigger particles the corresponding limit increases with increasing atomic number. This code can also simulate the effects of oxygen on the induction of clustered DNA lesions. Because of the fact that MCDS provides simulations tacitly for both direct and indirect DNA damage mechanisms, it uses an extraneous free radical scavenger (DMSO) and thus imitates diminutions in the total amount of strand breaks and base damages because of the scavenger’s presence [102]. It must be mentioned at this point that MCDS simulates the so called “initial” levels of DNA damage induced and not the processing or repair.

### 2.3. DNA Modelling

**Figure 3:**
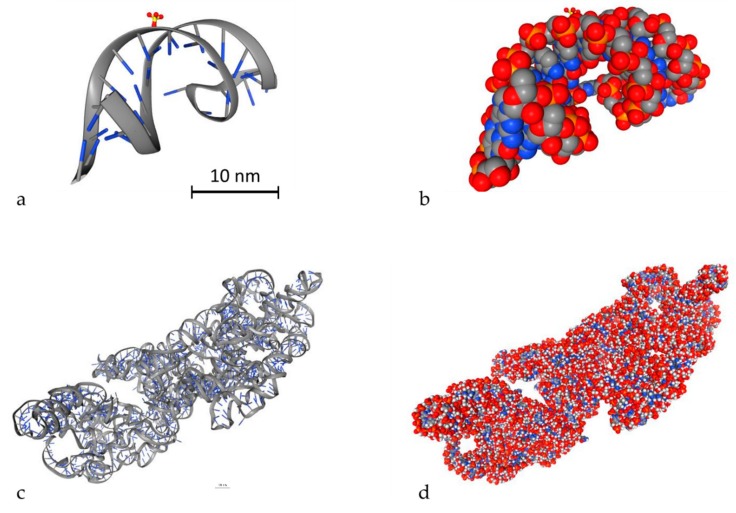
3D view of two different DNA molecules by the Proteins Data Bank (PDB) database. Scale bars have been added accordingly. Both scale bars represent a distance of 30 nm. (**a**) Simple small DNA, stylish view, (**b**) simple small DNA, “simulation” view, (**c**) complex DNA, stylish view, (**d**) complex DNA, “simulation” view.

An important parameter to simulate DDR is to model DNA molecules. There are two ways to model DNA molecules. One way is to use simulation techniques and the other one is to use geometry design techniques. There are also fixed DNA geometries that can be downloaded by the Proteins Data Bank (PDB) [116]. PDB (http://www.rcsb.org/) is a big database that contains high detailed DNA molecules, but it does not contain every DNA molecule. In Geant4-DNA, there is an example (PDB4DNA [117]) that includes a model by this database. In Figure 3, two different DNA molecules that were provided by the PDB database are presented in several styles. The stylish representation has been used to make the reader understand the geometry of the DNA molecule. The “simulation” representation is the one used by the MC simulation algorithms and they represent each different ingredient of the DNA molecule with spheres of different colors. Bases are shown in blue while the backbone is shown in grey and red. The spheres are the space where energy is deposited. The first one is a simple and small molecule (Figure 3a,b) (linear, with 20 base pairs length), while the second one (Figure 3c,d) is a more complex DNA molecule (5000 base pairs). Simulation software usually exploits the geometry shown in Figure 3b,d to calculate energy deposition.

A DNA simulation tool is MacroMoleculeBuilder (MMB) [118], which has been implemented using the SimTK simulation toolkit [119]. SimTK provides the user with several simulation tools, including Simbody (https://simtk.org/projects/simbody/) and Molmodel (https://simtk.org/projects/molmodel). These algorithms allow the implementation of specified joint constrains using coordinates, which allow the user to control the flexibility of the generated molecule. MMB can simulate the interactions between bases, which are exploited to produce the geometry of any combination of base pairs. An analytical classification of base pairs can be found in the Leontis–Stombaugh–Westhof catalog [120]. The catalog contains several combinations of force and torque that tend to produce an attachment frame around the first residue’s base, with a body frame on the second residue’s base.

Furthermore, Howell et al. proposed a simulation algorithm for the modelling of B-DNA [121]. They exploited coarse-grained (CG) simulations based on the bead-rod model reported by Wang et al. [122] that originated in the wormlike chain model [123]. In this model, the DNA molecules are designed using N beads connected by N−1 inextensible rods of length l, with a total length L. The final model has a length of L = (N−1)l. For energy compensation, the model employs a bending penalty between adjacent beads and an excluded volume repulsion between beads. This algorithm can generate several different structures where the only common thing is their ingredients. This model can only support homogeneous DNA bending and cannot be extended to more complex configurations that include DNA kinking and stretching [124]. 

Cumberworth et al. [125] introduced several sampling methods that create a model which can simulate computationally feasible DNA origami self-assembly. The model can contain structural parameters relevant to the process of construction. There are several different methods, using different sets of parameters, to produce similar designs. For large-scale systems, the results can be highly different as there is a connection between the variables of the origami design.

The above-described DNA simulation models have not yet been used in MC simulations. Recently, DnaFabric [126] has been presented and used in Geant4-DNA. The purpose of DnaFabric is to generate, edit, display, and export complex DNA geometrical models. It is based on a hierarchical geometry description designed to adapt to optimization techniques and adaptable level of detail (LODs). 

## 3. Monte Carlo Applications for Assessing Biological Damage 

Cellular response to IR has been investigated for years, showing the dependence of DNA damage on the deposited energy (usually linearly for doses up to at least 20 Gy), on the delivery time-frame, on the dose rate, on the radiation particle type and energy, and in general on the quality of radiation. Mechanistic simulations and mathematical modeling have been extensively used in biological and medical applications of IR. They simulate, estimate and quantify the absorbed dose, calculate the dose distribution, provide information on the radiation source and assess the biological effects of specified radiation. In addition, they help to integrate the knowledge acquired by the experimental data into a quantitative frame and finally to understand, analyze and inter-/extrapolate observed tendencies. 

### 3.1. Types of Irradiation techniques and Applications

**Figure 4:**
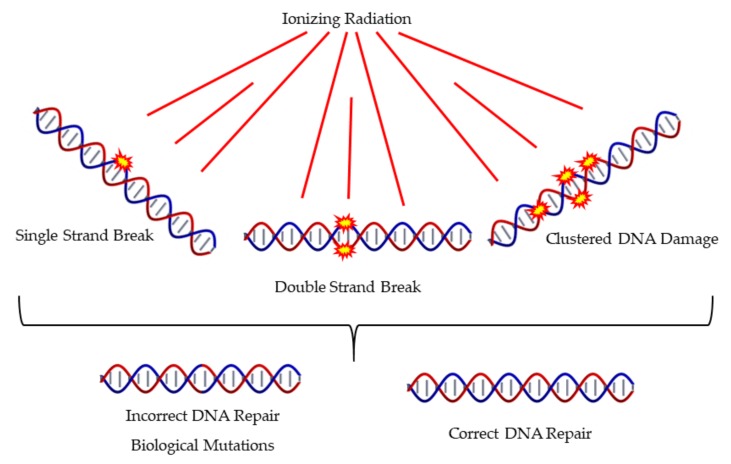
Induction of different types of DNA damage by ionizing radiation includes single and clustered forms of damage. This may often lead to misrepair.

The literature proposes that the main determinant of detrimental effects of IR is the complex DNA damage as presented in Figure 4, which also presents different mechanisms of DNA Damage and Repair. The nature of clustered DNA damage that is induced mainly by high-LET radiations has the ability to trigger biological pathways that differ from those induced by the everyday oxidative stress. This has been also supported by MC simulations [6]. Accumulating evidence, as presented in a recent review article by Mavragani et al. [127], shows that high-LET radiation usually triggers a very different biological response compared to low-LET IR like X-rays or γ-rays. Recent data by Jacob et al. suggests that even within the ion track core and the off-track δ-rays (high-energy electrons), there is a substantial difference in the complexity of damage, for example DSBs [128]. The core DNA lesions are difficult to repair and show a delay compared to the off-track δ-ray induced DSBs which rather resemble in complexity the ones induced by X-rays.

Several types of IR are exploited in medical science, either for low-dose procedures (usually imaging) or for higher doses (usually therapy), from X-rays and gamma rays to electron, protons and heavier ions, with a variety of dose rates. As already noted, they are used because of their special interaction with biological matter i.e., photons experience photoelectric effect and Compton scattering as well as pair production and Rayleigh scattering, while charged particles interact through other mechanisms. In general, the damage that is produced by the IR is categorized into two mechanisms, direct and indirect damage. 

Direct damage is produced when primary particle or secondary electrons, which have been produced by the interaction of primary radiation with matter, interact electromagnetically with tissues, cells, and the DNA. Indirect damage occurs when free radicals (e.g., OH^−1^), produced by the electromagnetic interaction of radiation with matter, interact chemically with tissues, cells, and DNA. Moreover, IR can also be categorized by the energy loss of radiation per unit path length, called linear energy transfer (LET), which is an indicator of the resulting biological effect, to (a) high-LET and (b) low-LET radiation. It must be noted that some of the work with radiobiological modelling at the nanometer scale is to search for a way to characterize IR other than LET. It is important to point out that even though LET is widely used, it has limitations when it comes to characterizing IR in terms of biological outcome. More specifically, we refer to the shortcomings of the relative biological effectiveness (RBE) vs LET relationship due to broad LET distribution, as in a single spread-out Bragg peak (SOBP) or in multiple overlapping radiation fields as discussed in [129].

### 3.2. Direct Damage Studies

When using Monte Carlo simulations, the best way to maximize accuracy is to make them as simple as possible. Thus, the first step to quantify DNA damage is to simulate only the physical part of IR interactions with matter. Several studies follow that path and model the direct damage produced by IR to the DNA, to guide the scientific community to a better understanding of the mechanisms involved. The studies that follow have made a basic assumption that must be stated, in that every part of the DNA molecule is supposed to be liquid water. 

Semenenko et al. [102] calculated the clustered DNA damage yield using the MCDS code and then they compared it with detailed Monte Carlo algorithms (MCTS codes). They concluded that MCDS is a very fast and user-friendly way to generate random DNA damage, because of the fact that its results reproduce the expected trends at least for secondary electron energies above ~0.5 keV [40]. Moreover, they explained that the good correlation between MCDS and detailed Monte Carlo simulations is an indication that the DNA damage is the result of independent and purely stochastic events and processes.

Huang et al. [130] used FLUKA and MCDS to estimate relative biological effectiveness (RBE) values for the induction of DSBs. FLUKA was used to estimate the absorbed doses and the fluence energy spectra of a high-energy carbon-ion beam in a water phantom and the MCDS to estimate DSB yields. 

Stewart et al. [131] used MCNP linked with MCDS to simulate DSB RBE for photons, neutrons and light ions. MCDS was also used to generate that DNA damage, but it was optimized with the insertion of RBE corrections produced using the MCNP code. They concluded that the combination of MCDS and MCNP, which was suggested in this work, did not require specification of any purely empirical parameters for the estimation of the RBE for initial DSB formation at any level (cellular, multi-cellular or tissue). These empirical parameters were implicitly included in MCDS through using previous results generated by MCTS.

TOPAS-nBio was validated by McNamara et al. [92] in comparison with experimental data and simulation results [132]. This extension of Geant4-DNA correlates detailed track information with realistic biological volumes. They claim that this is a very powerful and easy-to-use tool for the development of advanced radiobiology Monte Carlo Simulations.

Chatzipapas et al. [42] quantified DNA DSBs that were produced when a DNA molecule was irradiated by a LINAC, using Geant4-DNA. They compared simulation results with experimental data that were produced in parallel with this work, using a prototype dosimeter described in this study [133]. Their results showed good agreement between the simulation and the experiment.

### 3.3. Water Radiolysis: Indirect Damage Studies

The behavior of biological systems is under research in recent years by many groups trying to combine experimental knowledge with quantitative modeling to boost the understanding of both the physical and chemical interactions of cellular networks and also of the chemical networks of single cells [134,135,136,137]. However, there is still a long way towards the whole-cell simulation, which is a venture for computational biology [138]. 

Nikjoo et al. [139,140] used the MCTS code PITS [141] to generate primary-ion interactions (proton and alpha-particles) and CPA100 [142] to generate electron tracks, with initial energies from 10eV up to 100 keV, while generating the ensuing chemical tracks. They compared their results with experimental data for the yield of SB and the ratio of SSB/DSB. They also showed that the lengths of damaged sections of DNA tend to be quite short. The yield of strand breaks (SB) per unit dose is nearly constant over a wide range of LETs. SSBs are more likely to happen in comparison to DSBs, but complex SSBs appear in high frequency. 

Friedland et al., in a very extended article [40], simulated the interaction of high-energy particles (0.2–256MeV/u) with biological tissue, taking into account physical, physico-chemical, and chemical interactions. Indirect effects were assumed to arise from 65% of the interactions between free radicals and the DNA. Additionally, 1% conversion probability from an SB to a DSB has been assumed for both directly and indirectly induced breaks [143]. They calculated the LET for several types of particles (H, He, C, N, O, and Ne) and then evaluated the dependence of DNA SB with LET. The calculated SBs and DSBs included both direct and indirect effects of the radiation. They concluded that the yields of indirect SB decrease with increasing LET, while the directly induced SB remain constant and then also decrease at very high LET. They explained that the rise of radical production leads to their interaction with themselves (recombination), while leaving DNA to interact with what is left of them. For the decrease of direct effects at very high LET, they explained that the extra energy is wasted at the same breakage that has already happened. It must be noted that the results were depicted in terms of SB, DSB, DSB sites, DSB clusters, DSB number per cluster, and DSB from DNA fragmentation analysis. They also studied the effects of (Z_eff_/β)^2^ to DNA strand breakage and the DNA fragmentation dependency to LET. Their results were compared with published data by the fast Monte Carlo damage simulation (MCDS) tool [103,104,131]. 

Karamitros et al. [144] presented methods and models implemented in Geant4-DNA for the simulation of chemistry interactions. Based on the Smoluchowski reaction model, they extended the chemistry module which was initially described in [113] to reduce the computational complexity. In their method, time steps were dynamically computed as described in [145], and the user can choose to limit time steps to save computational time by avoiding multiple unnecessary small steps. The new approach takes account of the distance to check pre-selected reactants and to finally implement the k–d tree data structure [146]. With this technique, the closest pair of reactants for a specified reaction is found as well as the nearest neighbors that exist in a defined range.

Meylan et al. [87] simulated, in Geant4-DNA, the early DNA damage on a fibroblast cell nucleus produced after the irradiation of the cell. In this work, strand breaks on the DNA double helix produced by the interaction of IR with water in a direct and indirect manner were calculated. The DnaFabric software was exploited to design and produce the geometry of the cell nucleus model that was investigated. Primary protons of several energies ranging from 0.2 to 20 MeV were used. The resulting DNA double strand breaks agreed with quantification experiments that were provided by the literature and exploited the technique of pulsed field electrophoresis. They defined 17.5 eV as the deposited energy in the backbone of a nucleotide that is needed to produce a direct strand break (SB). Moreover, the indirect SBs were calculated by measuring chemical reactions that involved (a) OH- and a sugar (phosphate and 2-deoxyribose) and (b) OH- and DNA. Only a percentage of the aforementioned reactions could give SBs, 40% and 11%, respectively [147,148]. As stated in this study, the chemical stage, for simulation purposes, lasted 2.5 ns. To measure a DSB at least two SBs should be separated in distance by at most 10 bp and located on opposite strands.

Tang et al. [149] simulated by the full Geant4-DNA chain (that includes the physical, physico-chemical, and chemical stages) the IR action to endothelial cells by different photon energy sources. The IR-induced damage was quantified in terms of early DNA damage, specifically DSB induction, and the theoretical analysis included both microdosimetric and nanodosimetric methods. The damage complexity of DSB was also investigated and compared to experimental data expressed as γ-H2AX foci per cell. Fair agreement between simulated and experimental data was found. The simulation results helped explain the observed RBE variation between the kV and MV X-ray sources, highlighting the role of low energy secondary electrons with energies below 10 keV.

A theoretical framework was presented by Cuhna et al. [150] that can be used to calculate cell survival after a particle therapy procedure, named NanOx. NanOx is a multiscale model that integrates physical and chemical aspects of the radiation–matter interaction. The results were compared with experimental data and showed good agreement. This study was extended by Monini et al. [151], who made a theoretical study using different sets of parameters.

Recently, Tsai et al. [152] and Lai et al. [153] presented a novel, open-source, GPU-based MC simulation tool for the calculation of DNA damage produced by IR. The tool is based on Geant4-DNA physics lists but it can accelerate the simulation up to ~540 times, using an advanced GPU graphics card. The tool was validated and achieved similar accuracy to Geant4-DNA. It includes physical, physico-chemical, and chemical phases of the interaction of radiation with a cell nucleus and its DNA. It uses specific models of cell nucleus and DNAs and at this time it only accommodates electrons as IR. It only includes cross section for water, as it shares the physics lists of Geant4-DNA, thus everything in the geometry is considered to be water.

### 3.4. DNA Damage Repair Simulation Techniques

A very important parameter that must be considered when trying to simulate the DNA damage induced by IR is that the cell has the ability to repair itself. In most simulation codes, it is difficult, because of the time barrier, to take into account this fact, in parallel with the DNA damage calculation. However, there are a few studies that have calculated this variable.

Friedland et al. [58,59] have produced this kind of multiparameter study. PARTRAC was used for the biophysical MC simulations, where IR was interacting with liquid water, which was a substitute for cells and cell nucleus. A 3D model of a human fibroblast, including chromosomes with their kinetics and dynamics, was used as the DNA target, where SSBs and DSBs were scored by superposition of the energy deposition events. Chemical interactions were also taken into account, using specific probabilities. Four different scenarios were implemented for the repair process that used different assumptions. Experimental data were also used to adapt several parameters of the model. Chromosomal aberrations and mis-rejoined DSBs were found to be in good agreement with experimental data, while three scenarios overestimated residual DSBs after long-term repair, for low-dose irradiation procedures. 

Barnard et al. [154] explained in detail that even though DSBs are thought to be in linear correlation with dose, they are not. Some factors that explain this kind of non-linearity are, on the one hand, problems during the several different types of measurement and, on the other hand, the repair process of the DNA radiation-induced damage.

In a very preliminary study, Warmenhoven et al. [155,156] investigated, using simulation tools, the mechanisms in NHEJ. They developed a model that takes into account several mechanisms that are experimentally supported and were incorporated into a theory of sequential joining, to finally reproduce experimental results that are reported in the literature. Their results suggested that the motion of individual DSBs plays a crucial role in the repairing process and its kinetics. This model was incorporated into Geant4-DNA to provide the scientific community with the ability to extend physical interactions to the biological realm.

There have been groups that have only studied the modeling of the repair processes after the production of DSBs. A very preliminary study was one by Cucinotta et al. [157] that analyzed dosimetry and bio-dosimetry data of 19 ISS astronauts. Astronauts underwent bio-dosimetry assessment of chromosomal damage in lymphocyte cells. A mathematical model was presented that can simulate the mechanisms that are initiated after the radiation-induced DSBs. The model showed good correlation with astronaut data in organ doses as well as in chromosomal aberrations.

Taleei et al. [158] developed a mathematical model to predict the kinetics of DSB repair, for damage induced by high-LET radiation. Cells assumed to be in G1 and early S phase and DSBs were repaired with slow kinetics. The DSB repair mechanisms that were simulated were nonhomologous end-joining (NHEJ), homologous recombination (HR) and microhomology-mediated end-joining (MMEJ). Their calculations agreed with published experimental data of cells of Chinese hamsters and human dermal fibroblasts.

Rahmanian et al. [64] described a mechanical model of base excision repair (BER) for SSB repair. Published experimental data were used to validate the outcome of this model, which included irradiation with γ-rays, Si and Fe ions. Good agreement was achieved for both overall repair and the repair of complex lesions.

Woods et al. [159] presented a new method to model the NHEJ, single strand annealing, and alternative end-joining mechanisms that are initiated after exposure to IR. The model was validated using biological experimental datasets and Bayesian statistics. The results showed that DNA dynamics for repair processes could be included in one theoretical framework.

Cleri et al. [160] simulated microsecond-long molecular dynamics for the repair of nucleosomes that contained DSBs. The results showed that broken DNA ends remain attached to the nucleosome and they need to overcome an energy barrier to be detached. 

Tello Cajiao et al. [161] extended the results of the Monte Carlo code called biophysical analysis of cell death and chromosome aberrations (BIANCA), for intermediate- and high-LET radiations. Both studies showed that (mis-)rejoin probability depends on the initial distance of the two DNA fragments, radiation quality, cell type, and dose.

Recently, Li and Cucinotta [162] developed a mathematical model to study the dynamics of ataxia-telangiectasia mutated (ATM), a mechanism that is triggered when cells are irradiated with IR and DSBs produce rapid local chromatin relaxation. The outcome was that the model could predict ATM-mediated responses to DSBs, which can be evaluated with experiments.

### 3.5. DNA Damage Quantification Techniques Other Than Monte Carlo

Apart from these Monte Carlo codes, which attempt to describe mechanistic DNA damage and repair kinetics, there are also many radiobiological phenomenological macroscopic models which have been developed to describe the response of cells under irradiation, which correlate cellular reaction to the delivered dose and other parameters expressing cell sensitivity, and which are currently used in clinical practice for treatment planning. The most common example is the linear quadratic (LQ) cell survival model [163] which provides a simple relationship between cell survival and delivered dose and is widely used both experimentally and clinically [164]. For getting a better interpretation of high-dose survival feedback (especially in stereotactic radiotherapy), several models have been suggested, such as the Padé linear quadratic (PLQ) model [165], the universal survival curve (USC) model [166] and a mechanistic formulation of a linear-quadratic-linear (LQL) model in the case where we have split-dose experiments and exponentially decaying sources [167], which all contribute to clinical applications. Other well-known models include the local effect model (LEM) which describes biological effects based on amorphous track structure in conjunction with dose response after X-ray irradiation [96,168], and the microdosimetric kinetic model (MKM) which uses estimations of stochastic energy deposition into volumes of the μm-scale [169]. Other approaches use macroscopic simulations to achieve realistic radiation fields, such as the multiscale approach (MSA) which calculates the probability of survival for cells that have been irradiated with ions, taking into account the differences in each situation, such as the space, energy, and time of the experiment [170]. Furthermore, the mechanistic model BIANCA provides, in addition to cell survival curves, chromosome aberration dose-response curves, describing the interphase chromosome organization as well as the connection between chromosome aberrations with cell death [171]. Of the same class, the repair-misrepair-fixation (RMF) model has been developed to link double-strand break (DSB) induction to reproductive cell death [172]. The mechanistic model proposed by Wang et al. offers predictions at the molecular and cellular levels that are quantitatively described by only two input parameters [173]. A general comment on all existing models can be that one can argue that LEM and MKM are not purely phenomenological, but rather mechanism-inspired models. The theory behind them is highly mechanistic even though in the end they may fit parameters to the published experimental data including the RMF model. Purely phenomenological models are those based on fitting parameters to obtain LQ alpha and beta values as a function of LET [174].

There are also other approaches which extend research further down in scale, such as MBN explorer which models the interactions between molecules [175]. 

Recently, the RITCARD (Radiation Induced Tracks, Chromosome Aberrations, Repair, and Damage) algorithm was introduced, which even though it is not a MC code can model the human chromosome geometric configuration as well as simulate the radiation-induced breaks and their repair, which lead to various chromosome aberrations [176,177]. RITCARD is based on a repair kinetics model and was created as an extension beyond the original applications of the NASA code radiation track image (NASARTI). This program has been specially developed to estimate chromosome damages that astronauts will incur from exposure to high-LET space radiation, and to assess cancer risk of future deep space missions.

## 4. Conclusions and Future Prospects

This study reviewed several Monte Carlo techniques incorporating DDR to ionizing irradiation. As demonstrated in this review, the need of accurate techniques for RBE quantification is crucial, as it becomes a clinical need to evaluate DDR for a variety of applications including both low- and high-energy radiation. Scientific interest for radiobiology studies using Monte Carlo methods has been continuously increasing over the last twenty years, providing great perspectives in the field of radiation physics. Radiation transport codes and MCTS codes have been used to simulate DNA damage induction due to IR. For charged particle radiation, there are few experimental data available, and therefore MC codes have proved to be a unique but sophisticated tool for the satisfactory qualitative and quantitative interpretations of the experimental data. These codes contribute a lot to our understanding of the underlying action mechanisms of the radiobiological interactions on the cellular level. They are based on simulation of stochastic processes using random number generators.

The DDR network includes pathways of high complexity that represent obstacles towards the optimization of cancer treatment. A better understanding would undoubtedly lead to further improvement on the therapeutic clinical protocols. There is still a very long way to go to model DDR to a similar level of detail as has been done for damage induction, and an even longer way before the results might be used in medical applications. A better understanding on the DDR mechanisms would identify synthetic lethal relationships that could be exploited to improve the cancer therapy in general, and also to develop personalized therapies based on patient-specific DDR of tumors [178,179,180,181]. Furthermore, using this knowledge it could be made possible to predict any change in the DDR of different tumor species and modify accordingly general and personalized therapeutics. This way, oncologists could monitor tumor response to the initial therapy and when there is a resistance, a more aggressive technique may be exploited or a different therapy pathway be taken. Thus, it would be proved that cancer cells include genetic mutations that confer resistance prior to treatment [182,183].

To summarize, in this review the challenges and advances in the detection, modelling, simulation, and correct estimation of the biological importance of IR-induced DNA damage are presented. Detection ventures are considered the most severe obstruction towards the establishment of definite relationships between the damage induced by IR and the prediction of biological reactions on the DNA level, but more specifically, going a step further to the organ level, studying groups of cells.

Monte Carlo simulations are a well-established tool for use in radiobiology as there are clear prospects for developing more advanced tools that could be used in multidisciplinary studies involving physics, medicine, biology and chemistry. Still, lots of effort is needed to evolve simulation tools and to be able to apply them on studies of cells and cell population (tissues) or even (in the far future) on human organs. There is a clear need for more experimental data that may quantify DNA damage and study the DDR. Despite the current limitations, MC techniques seem to provide a very promising tool, incorporating recent advances in computing science that could even lead to simulations of personalized radiobiological studies during IR medical applications.
